# Exploring a Cadaver-Based Model for Teaching Emergency Medicine Residents Ultrasound-Guided Serratus Anterior Plane Blocks

**DOI:** 10.7759/cureus.45178

**Published:** 2023-09-13

**Authors:** Brandon M Wubben, Michael R Wallum, Cory A Wittrock

**Affiliations:** 1 Department of Emergency Medicine, The University of Iowa, Iowa City, USA

**Keywords:** ultrasound-guided procedures, cadaveric study, point-of-care ultrasound (pocus), emergency medicine training, ultrasound-guided regional anesthesia, ultrasound-guided, fascia iliaca compartment block, serratus anterior plane block

## Abstract

Background

Ultrasound-guided regional anesthesia (USGRA) is increasingly being incorporated into ED clinical practice to provide pain control for a variety of traumatic injuries. The serratus anterior plane block (SAPB) has been shown to be effective at reducing intravenous opioid use and improving pulmonary function for patients with rib fractures, but there is limited prior research about how to safely teach this procedure to emergency medicine (EM) residents. Our goal was to examine the effect of a cadaver-based education model on EM residents’ confidence in performing USGRA and provide a review of commonly encountered errors.

Methods

EM residents participated in a half-day cadaver-based education session that included a variety of less-commonly performed procedures including SAPB and fascia iliaca compartment block (FICB) USGRA. Residents received a didactic lecture and hands-on simulation practice during the month prior to the session. During the session, residents performed a SAPB and FICB on the cadaver patient using the same nerve block kit and ultrasound machine they would use for a living patient, with 1:1 supervision by an emergency ultrasound fellowship-trained physician who provided real-time feedback during the procedure. Representative ultrasound video clips were saved and reviewed. Surveys that were completed by residents after the session were analyzed.

Results

There were 23 residents who participated, and most had not performed any FICB (74%) or SAPB (87%) previously. The percentage of residents comfortable with general USGRA increased from 8.7% to 91.3% (p<0.001). Comfort with FICB increased from 9.1% to 77.3% (p<0.001), and comfort with SAPB increased from 9.1% to 77.3% (p<0.001). Instructors identified and corrected several common errors, such as overly aggressive needle insertion, instillation of air, and instillation of anesthetic into muscle rather than the fascial plane.

Conclusion

We found that a cadaver-based education model for teaching EM residents the SAPB and the FICB was associated with significant increases in resident confidence in performing the procedure and facilitated identification and correction of common procedural errors that may otherwise have gone undetected.

## Introduction

Rib fractures are a common and painful finding in injured trauma patients, often requiring intravenous opioids and hospital admission [1]. Following initial case reports of providers successfully using serratus anterior plane blocks (SAPB) for rib fracture analgesia [2], two prospective cohort studies and a randomized controlled trial demonstrated similar success [3-5]. These studies found that the SAPB reduced intravenous opioid use and improved pulmonary function following the block. Hip fractures are another common injury requiring pain management strategies, and utilizing the fascia iliaca compartment block (FICB) has been shown to decrease pain, opioid use, and hospital length of stay [6,7]. The cadaver model has previously been shown to be successful in teaching other ultrasound-guided blocks, such as forearm and brachial plexus blocks [8], but has not been explored for serratus anterior.

The goal of our study was to explore a cadaver-based education model to teach emergency medicine (EM) residents how to perform ultrasound-guided SAPB and FICB. We evaluated the effect of this training on resident confidence in performing these procedures and provided a video review of common errors.

## Materials and methods

This was a prospective, single-center study at an academic medical center with a three-year EM residency. Our EM residency program holds an annual cadaver-based education session focused on teaching procedures performed less frequently in clinical practice. There were 28 physician residents and four advanced-practice providers in the residency program; not all residents were able to attend due to a portion of residents being on elective rotations and vacation. For the Spring 2023 session, two of the procedures that were included were the ultrasound-guided FICB and the SAPB.

An introductory lecture about the basics of ultrasound-guided regional anesthesia (USGRA) was given during resident didactics during the month preceding the cadaver-based education, which covered basic knowledge about USGRA including the risks of local anesthetic toxicity, equipment setup, and patient positioning. A separate hands-on session was taught in the weeks preceding the cadaver lab, with residents scanning live volunteers to identify the relevant anatomy and practicing live needle guidance on ultrasound phantoms. On the day of the cadaver-based education, residents were divided into small groups to allow every resident to perform the procedure with an emergency ultrasound fellowship-trained EM physician instructor with USGRA experience, with an instructor-to-participant ratio between 1:1 and 1:3. The ultrasound platform used for hands-on practice was the Sonosite PX (FUJIFILM Inc., Bothell, WA). The same supplies that are available for clinically performed USGRA in the ED were provided for this session.

Post-session surveys were distributed to record the level of training, prior experience, perceived changes in comfort level with USGRA and each block type, perceived likelihood of USGRA use in clinical practice, and opinions about the teaching format (Appendices). Descriptive statistics were analyzed. Before and after paired categorical survey responses were compared using the X2 McNemar test, with "very comfortable" and "somewhat comfortable" binned as "comfortable" and "very uncomfortable" and "somewhat uncomfortable" binned as "uncomfortable" for dichotomous analysis. SPSS Statistics version 28.0 (IBM Corp. Released 2021. IBM SPSS Statistics for Windows, Version 28.0. Armonk, NY: IBM Corp) was used for statistical analysis. This project was granted exempt status for written informed consent by the University of Iowa Institutional Review Board (approval no. 202305256).

## Results

There were 23 residents who attended the session and completed the survey, out of 32 total residents in the program (71.9%). Most residents reported receiving prior education about USGRA, including web resources and textbooks (73.9%), departmental lectures (69.6%), prior hands-on instruction (65.2%), and bedside clinical instruction (43.5%). However, most residents (91.3%) had performed five or fewer ultrasound-guided nerve blocks previously, and most had not performed any FICB (73.9%) or SAPB (87.0%).

**Figure 1:**
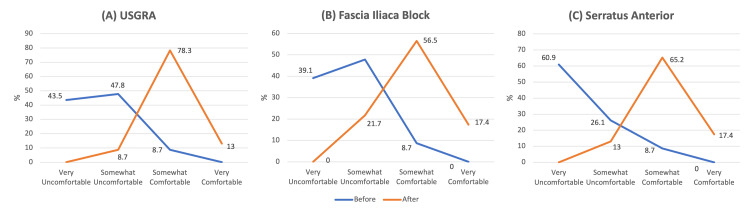
EM residents’ self-reported confidence in performing (A) USGRA, (B) ultrasound-guided FICB, and (C) ultrasound-guided SAPB before and after the cadaver education USGRA: ultrasound-guided regional anesthesia

**Figure 2:**
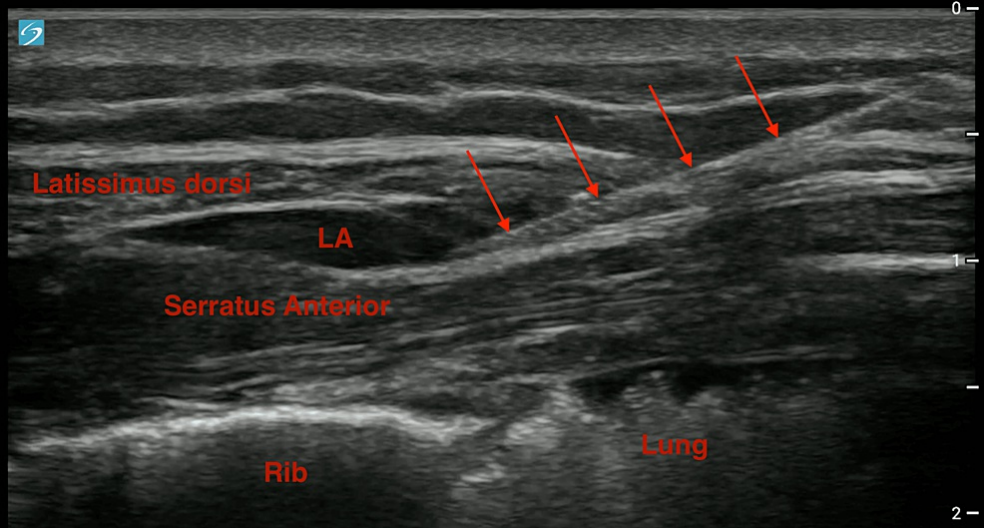
SAPB in progress, performed by an EM resident on a cadaver patient, with arrows showing the needle path LA: local anesthetic

The percentage of residents comfortable with general USGRA increased from 8.7% to 91.3% (p<0.001), the percentage of residents comfortable with FICB increased from 9.1% to 77.3% (p<0.001), and the percentage of residents comfortable with SAPB increased from 9.1% to 77.3% (p<0.001) as shown in Figures 1A-1C. Representative images of the SAPB being performed are shown in Figure 2. Most (95.5%) of residents reported they were very or somewhat likely to use FICB and SAPB in their future practice. All of the residents reported that the ultrasound education was useful and that the education was additive to their knowledge of USGRA procedures.

Although instructors had previously performed other USGRA techniques using a cadaver patient, they had not previously tested the SAPB. Instructors tested the model themselves and felt that the experience of performing SAPB and FICP on cadaver patients realistically captured the experience of performing these blocks on living patients.

A successful SAPB is shown, starting with a test injection of 1-2 cc local anesthetic that spreads easily along the fascial plane as a thin hypoechoic line (Video 1). A successful test injection is followed by a higher volume injection (shown on a different cadaver patient) to complete the block (Video 2). Instructors witnessed several errors that were able to be corrected, including overly aggressive needle insertion, which risks complications such as pneumothorax if the needle is inserted too deep and violates the pleura (Video 3). Providers should stabilize their needles by anchoring their hands against the patient in such a way that the needle is not allowed to rush forward after penetrating the skin. Inadvertent injection of air, which obscures the field of view, is easily corrected by priming the tubing and needle with local anesthetic prior to inserting the needle (Video 4). Inadvertent injection of local anesthetic into the muscle is a common error and one of the reasons a test injection is needed prior to large-volume administration. In this example, the needle should be withdrawn slightly to ensure instillation in the fascial plane (Video 5). Instructors felt that the ability to address these errors prior to residents performing the procedure on living patients was qualitatively invaluable.

**Video 1:** A successful SAPB test injection. A thin, hypoechoic line of local anesthetic spreads easily along the fascial plane

**Video 2:** A successful SAPB. Local anesthetic spreads easily along the fascial plane, superficial to the serratus anterior muscle

**Video 3:** A SAPB with overly aggressive needle insertion, which risks complications such as pneumothorax

**Video 4:** A SAPB with an inadvertent injection of air

**Video 5:** A SAPB with inadvertent injection of local anesthetic into the muscle instead of the fascial plane

## Discussion

We found that cadaver-based education for teaching EM residents SAPB and FICB was associated with significant increases in resident confidence in performing these procedures and facilitated identification and correction of common procedural errors.

Teaching USGRA traditionally includes didactics and a simulation-based model, followed by supervised performance on living patients, but it is unclear if this approach translates well to USGRA use in patient care [9]. Cadaver patients offer trainees an additional stepping stone to learn USGRA before performing blocks on a living patient. A prior scoping review found that cadaver-based education was relatively infrequently used in resident ultrasound education [10]. However, some studies have shown cadaver-based models to improve resident confidence in performing forearm, brachial plexus, and fascia iliaca nerve blocks [8,11].

Residents in our study reported through informal feedback that the tactile sensation of performing USGRA on a cadaver patient was superior to traditional simulation-based models. In addition, the cadaver patient could be positioned in the same way as a living patient, and finding anatomical landmarks with ultrasound was far superior to any simulation model. With the supervision of an expert in USGRA, learners reported benefiting from real-time feedback on their technique. The instructors in our study were able to identify and correct several procedural errors that may not have been identified using commercial or homemade simulators and otherwise may not have been discovered until residents started supervised practice on living patients.

To our knowledge, teaching SAPB using the cadaver model has not previously been studied, and the current study adds to the literature supporting the use of cadaver-based education for USGRA. Future studies might examine whether the addition of cadaver-based models to resident education translates to increased use of USGRA in clinical practice, decreases complications, or reduces time to procedural mastery.

Limitations

Although residents reported increased confidence in performing USGRA after cadaver-based education, we did not examine objective measures of resident competence or changes in clinical practice. No previously validated survey instrument was available, although the structure was based on prior ultrasound education surveys. This was a single-site study, and the benefits of a similar education program at another institution may vary depending on baseline USGRA experience, which was low among our residents.

## Conclusions

Training EM residents to perform SAPB and FICB using a cadaver-patient education model was associated with increased resident confidence in performing these procedures. Residents uniformly reported the training to be useful and additive to their knowledge of USGRA. Informal feedback highlighted benefits such as the ability to use real-world patient positioning and the ability to experience needle haptics in real tissue. Instructors were able to identify and correct several common errors through direct observation, such as overly aggressive needle insertion, instillation of air, and instillation of anesthetic into muscle rather than the fascial plane. Instructors felt that the opportunity to correct these errors prior to residents performing SAPB on living patients was qualitatively invaluable.

## Figures and Tables

**Figure 3:**
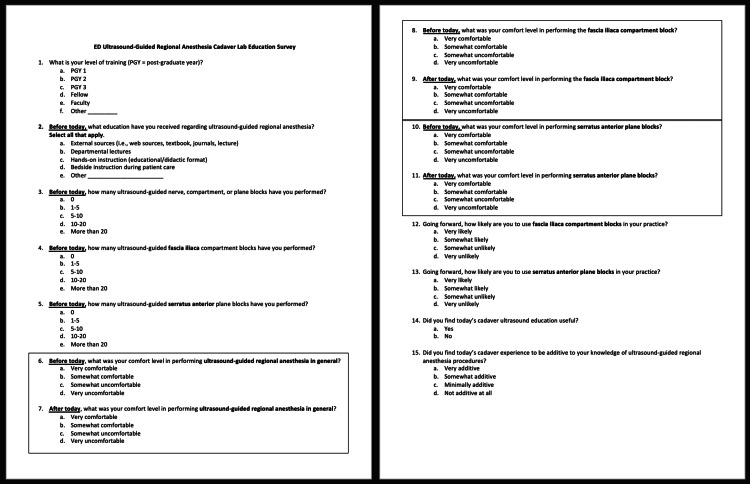
Ultrasound-guided regional anesthesia resident survey
